# Analysis of Indium Oxidation State on the Electronic Structure and Optical Properties of TiO_2_

**DOI:** 10.3390/ma11060952

**Published:** 2018-06-05

**Authors:** Matiullah Khan, Zhenghua Lan, Yi Zeng

**Affiliations:** 1 State Key Lab of High Performance Ceramics and Superfine Microstructure, Shanghai Institute of Ceramics, Chinese Academy of Sciences, Shanghai 200050, China; matiullahustb@gmail.com; 2 Shanghai Career Metallurgy Furnace Material Co., Ltd., Shanghai 201908, China; lanzhenghua716@163.com; 3 Department of Physics, Kohat University of Science and Technology (KUST), Kohat 26000, Pakistan

**Keywords:** Indium doped TiO_2_, oxidation state, doping concentration, optical response

## Abstract

Due to the high formation energy of Indium interstitial defect in the TiO_2_ lattice, the most probable location for Indium dopant is substitutional sites. Replacing Ti by In atom in the anatase TiO_2_ shifted the absorption edge of TiO_2_ towards visible regime. Indium doping tuned the band structure of TiO_2_ via creating In 5p states. The In 5p states are successfully coupled with the O 2p states reducing the band gap. Increasing In doping level in TiO_2_ improved the visible light absorption. Compensating the charge imbalance by oxygen vacancy provided compensated Indium doped TiO_2_ model. The creation of oxygen vacancy widened the band gap, blue shifted the absorption edge of TiO_2_ and declined the UV light absorption. The 2.08% In in TiO_2_ is the optimal Indium doping concentration, providing suitable band structure for the photoelectrochemical applications and stable geometrical configuration among the simulated models. Our results provide a reasonable explanation for the improved photoactivity of Indium doped TiO_2_.

## 1. Introduction

Environmental pollution and energy crises are the major problems attracting considerable attention from researchers. A semiconductor like Titanium dioxide (TiO_2_) could be used for environmental cleanup and renewable energy sources [1,2]. However, the wide band gap limits the efficiency of TiO_2_ in photoelectrochemical applications. With the help of Plasmonic nanoparticles, the limited absorption of TiO_2_ can be resolved [3,4]. However, for most practical applications, the usage of expensive metal nano-particles is not economically feasible. Doping foreign atoms in the network of TiO_2_ could be a suitable way to tune the band gap utilizing majors part of the solar spectrum [5,6]. The proper oxidation state of the dopant atoms can favorably stabilize the system and tailor the band structure. Inducing dopant atoms in any semiconductor materials disturbs the structure of the bulk material and the modification depends upon the difference in the ionic radii of bulk and dopant ions. Wide functionalities of the TiO_2_ could be induced by selecting a dopant with the proper oxidation state. Thus, the band structure of TiO_2_ could be tuned to utilize a major part of the solar energy with minimum structure distortion [7,8,9,10].

Introducing foreign atoms into the bulk of TiO_2_ either creates states in the forbidden region or mixes with the valence or conduction band modifying the electronic band structure [11,12]. Doping non-metal extended the absorption edge of TiO_2_ towards a visible regime by creating impurity states (in the forbidden region) or narrowing the band gap [13,14,15]. The states due to nitrogen (N) appeared above the valence band maximum which might annihilate the photo-generated carriers [16]. The efficiency of transition metals doped TiO_2_ is also limited due to the existence of localized d states and the recombination centers [17]. Existence of the isolated states in the band gap effect the optical absorption spectra and photo-activity of TiO_2_. Unoccupied isolated states often act as an electron trap, promoting the electron-hole pair recombination [18,19]. Removing the unoccupied states from the band gap or mixing it with the valence or conduction band would increase the lifetime of the photo-excited carriers, thereby improving the photo-activity.

Mixing the oxide of Indium (In) with TiO_2_ and improving the photo-activity are widely reported in literature. Nanocrystalline TiO_2_-In_2_O_3_ powders with different Ti/In ratios are prepared by sol-gel method and the photodegradation response is evaluated. Mixing In_2_O_3_ with TiO_2_ improved the photoelectrochemical properties of TiO_2_ [20]. The photoactive In_2_O_3_-TiO_2_ mixed oxides are extensively studied by Gonzalez et al. [21] and the photocatalytic activity are explored. The Indium oxide (In_2_O_3_) in combination with the silver improved the photoactivity of TiO_2_ [22]. Wang et al. [23] prepared Indium doped TiO_2_ by the sol-gel method and evaluated the photo-activity under visible light illuminations. Doping Indium in the TiO_2_ lattice improved the activity for the degradation of 4-chlorophenol under visible light irradiations. The topic of mixing the In_2_O_3_ with TiO_2_ or doping Indium in the structure of TiO_2_ is experimentally investigated. However, the alterations in the band structure and lattice of TiO_2_ due to Indium doping are rarely reported. To elucidate the effect of Indium doping on the geometrical structure, band structure and photo-response, it is suggested to perform some ab-initio calculations.

This work report DFT based calculations for Indium (In) doped TiO_2_ with different In doping level. Charge neutralization is made by generating oxygen vacancy and the electronic band structure and optical properties are evaluated.

## 2. Method of Calculations

With Materials Studio 8.0, the calculations are performed based on the plane wave method of DFT. The generalized gradient approximation (GGA) parameterized by PBE is utilized as an exchange correlation potential [24]. Keeping the maximum energy equal to 400 eV and k-mesh of 2 × 2 × 2, the electrons wave functions are expanded (in plane waves). The simulation environment is the ideal environment which is relaxed using some constraints for optimizing the simulated models. Using the BFGS minimization scheme [25], the maximum displacement was set to 5.0 × 10^−4^ Å, while the convergence limit for self-consistent tolerance was 2.0 × 10^−6^ eV/atom.

**Figure 1:**
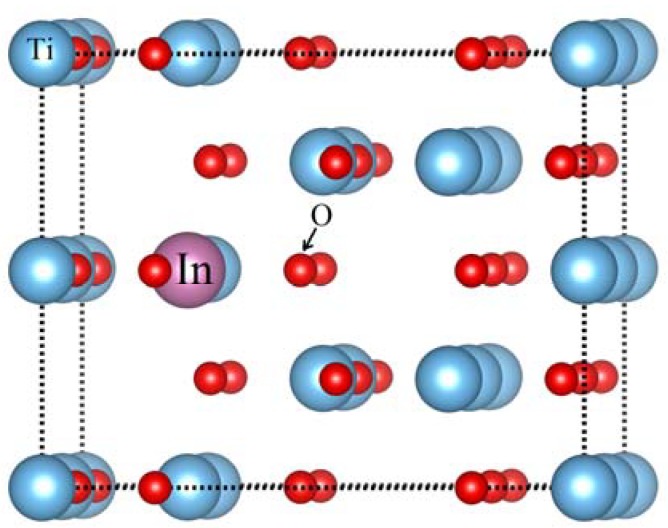
Insertion of Indium atom at Ti site in anatase TiO_2_ network.

Anatase TiO_2_ supercell is made from the replication of 2 × 2 × 1 TiO_2_ unitcell. Low concentration of Indium in anatase TiO_2_ are possible by increasing the size of the supercell. Indium (In) doped models are constructed by replacing the lattice Ti atoms by In atoms. Doping single In atom at Ti sites have the In doping level of 2.08% represented by Ti_16-1_In_1_O_32_. The doping concentration of In is increased to 4.16% and this system is named as Ti_16-2_In_2_O_32_. The effect of oxygen vacancy on the band structure is evaluated by creating one oxygen vacancy along with two In atoms at Ti sites and it is represented by Ti_16-2_In_2_O_31_. Figure 1 displays the atomic configuration of anatase TiO_2_ having substitutional In (at Ti site) and oxygen vacancy. As a standard model, a defect free bare TiO_2_ is also simulated.

## 3. Results and Discussion

The calculated lattice parameters for pure TiO_2_ (anatase) are; a = 3.81 Å and c = 9.71 Å, compared to the reported theoretical (a = 3.81 Å and c = 9.48 Å) [26] and experimental data (a = 3.78 Å and c = 9.49 Å) [27]. The bond lengths of the optimized systems are averaged and shown in Table 1. The O-Ti and O-O bonds are 1.9651 and 2.7034 Å, respectively. The Dmol^3^ based calculated Ti-O bond length for pure TiO_2_ is reported to be 1.930 Å [28]. Treacy et al. [29] studied the structural features of anatase TiO_2_ (101) with surface x-ray diffraction (SXRD) and then compared the results with the calculations based on density functional theory (DFT). It is found that the Ti-O bond length fluctuates between 1.89 ± 0.01 Å and 2.08 ± 0.01 Å [29]. Inducing defects modified the averaged bond lengths of the optimized TiO_2_. The bonds of Ti_16-1_In_1_O_32_ are stretched compared to the bare TiO_2_ which might be due to the replacement of lattice Ti by In atom. Increasing the Indium doping concentration further elongates the O-Ti bond length of the Ti_16-2_In_2_O_32_, however, the O-O bond length is reduced relative to the TiO_2_ and Ti_16-1_In_1_O_32_. Furthermore, the D_O-In_ of the Ti_16-2_In_2_O_32_ is also stretched with respect to Ti_16-1_In_1_O_32_. Oxygen vacancy has an interesting influence on the bond lengths of the doped models. With the same doping concentration, the D_O-Ti_ of the Ti_16-2_In_2_O_31_ is reduced while the D_O-O_ is elongated compared to Ti_16-2_In_2_O_32_. In addition, the D_O-In_ bond length showed no considerable variation. Modifications in the bond lengths of defect induced models are attributed to the different ionic radii of Ti^4+^ (68 pm) and In^3+^ (81 pm) [30,31]. Comparing the relative changes induced in the bond lengths due to different doping configuration, the Ti_16-1_In_1_O_32_ modelled system provided minimum structure distortion in reference to bare TiO_2_. This system might improve the stability of the doped TiO_2_ system and further improve the efficiency of In-doped in photoelectrochemical applications.

**Figure 2:**
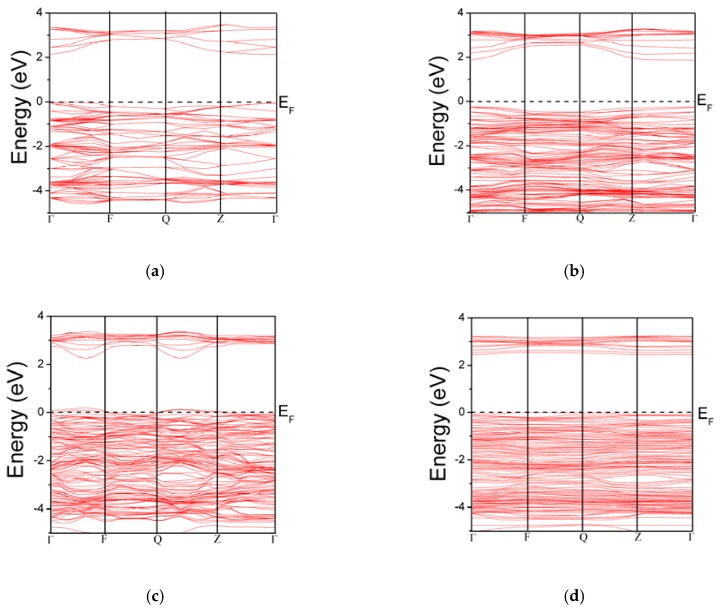
Band structure of TiO_2_ and defect induced models: (**a**) TiO_2_; (**b**) Ti_16-1_In_1_O_32_; (**c**) Ti_16-2_In_2_O_32_; and (**d**) Ti_16-2_In_2_O_31_.

The electronic band structure of the simulated models is depicted in Figure 2. The DFT based theoretically calculated electronic band gap is associated with the photoemission data. Similarly, the optical gap in the form of direct band gap could be linked with the spectrum of excitonic effects. The absorption spectrum of the TiO_2_ has a relationship with the optical band gap. Therefore, the spectrum of the TiO_2_ can be tuned by doping various elements, which create states in band gap or mix with valence or conduction band [5,32,33]. The calculated band gap of pure TiO_2_ (2.13 eV) is underestimated relative to the 3.20 eV. The underestimation is a drawback of GGA based calculations [34]. In case of known exact exchange-correlation potential, the DFT can only have access to the ground state properties. To deal with this problem, the electronic excitations modeled by Kohn-Sham equations should be substituted by the Dyson equation. Thus, the unknown exchange-correlation potential is modeled by an operator depending on the self-energy. The utilization of the Hubbard model along with DFT is one of the ways to deal with the underestimated band gap [10,35,36]. We consider the current study as a relative study that describes the relative changes induced due to different doping/defect configurations. So, the underestimation would not affect our results. Replacing the lattice Ti atom by In atom (Ti_16-1_In_1_O_32_), the band gap is reduced to 2.113 eV. The reduction in the band gap would be helpful in shifting the absorption regime towards visible light. Moreover, the Fermi level is moved slightly upwards in the forbidden region. The location of the Fermi level and the band edge positions of different models are summarized in Table 2. At gamma point, the band gap of Ti_16-2_In_2_O_32_ is 2.65 eV. Though the band gap seems to be extended, some humps are present at the conduction band maximum and it may help in migrating the electrons from valence to conduction band. Creating oxygen vacancy widens the band gap and the band gap for Ti_16-2_In_2_O_31_ is 2.483 eV. The widening of the band gap due to oxygen vacancy in TiO_2_ is consistent with literature and this increase might be explained by the localized states [37,38].

**Figure 3:**
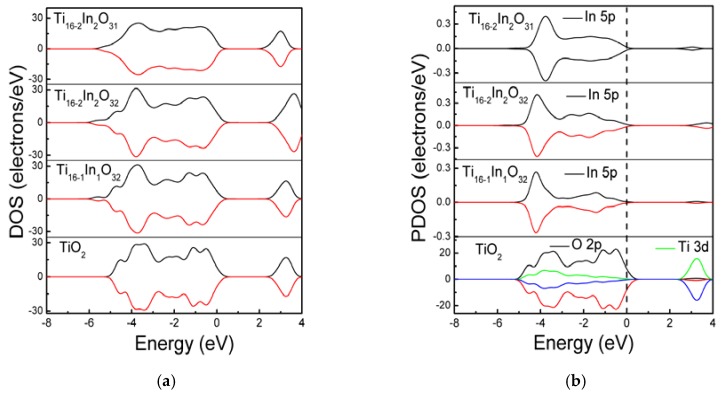
The (**a**) total density of states and; (**b**) partial density of states.

The band structure modifications are further investigated and densities of states are calculated. Figure 3 displays the density of states of the simulated models. Doping In atoms modified the band structure of TiO_2_ and the In 5p states are coupled with the host O 2p and Ti 3d states. Increasing the In doping level from 2.08% to 4.16% has no considerable effect on the band gap. Figure 3b demonstrates the partial density of states (PDOS) of the models. It is clarified that the valence band of bare TiO_2_ comprises of O 2p states while the Ti 3d contribute (predominantly) to the conduction band. Adding In atoms at Ti sites induced In 5p states which are coupled with the O 2p states, modifying the valence band of TiO_2_. In the Ti_16-2_In_2_O_32_ model, the Indium doping concentration is increased to 4.16% and the density of In 5p states near the valence band is enhanced. Moreover, some In 5p also contribute to modifying the conduction band. The intensity of In 5p states in the Ti_16-2_In_2_O_31_ model remains the same. However, the In 5p states are smoothened compare to the non-compensated systems. The In 5p states in the band structure would be supportive in migrating the visible light photons from valence to conduction band.

**Figure 4:**
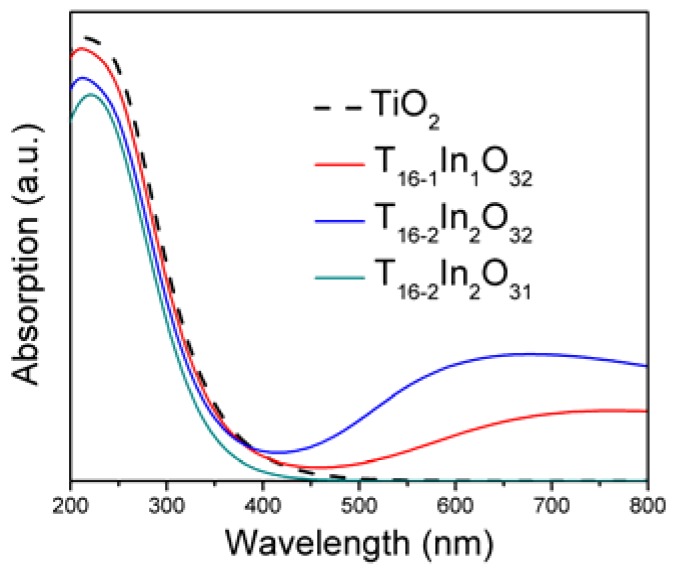
Optical response of the TiO_2_ and defect induced TiO_2_ models.

Figure 4 depicts the optical absorption spectra of the simulated systems. Tuning the band structure modifies the optical absorption properties of the semiconductor materials. For evaluating the optical properties, the underestimated band gap is updated to the experimental value of the band gap (3.20 eV) with scissor approximation [12]. The spectrum of bare TiO_2_ displays absorption in the UV region only because the wide band gap deprives it from utilizing the major part of the solar energy. The UV light absorption corresponds to the excitations of electron between O 2p and Ti 3d states. The In doping in the anatase TiO_2_ network shifted the absorption range towards the visible regime. It is clarified from Figure 4 that with the UV absorption, the Ti_16-1_In_1_O_32_ model also exhibits visible light absorption. The In 5p states are responsible for shifting the absorption threshold towards visible light region. In this case, the electron undergoes step wise transition from O 2p to Ti 3d states via In 5p states. Thus, it indirectly modifies the absorption spectra. Increasing the Indium doping level improved the absorption in the visible regime. The shifting of the absorption edge of TiO_2_ towards visible regime due to In doping is consistent with the reported data [23]. Inducing oxygen vacancy in the Indium doped TiO_2_ model made the absorption peak disappear in the visible light region. In addition, the Ti_16-2_In_2_O_31_ model only absorbs UV light, confirming the findings of band structure analysis. Furthermore, the UV light absorption is also reduced compared to bare TiO_2_. The Ti_16-2_In_2_O_31_ blue shifted the absorption regime of the TiO_2_. Stable structure, favorable band structure and improved optical response of In-doped TiO_2_ would improve its efficiency in photoelectrochemical applications.

## 4. Conclusions

Substitutional In doping at Ti sites reduced the band gap of anatase TiO_2_ and shifted the absorption edge toward the visible regime. Along with the absorption in the visible light region, the absorption in UV regime is also improved in reference to pure TiO_2_. The band structure of TiO_2_ is modified due to the creation of In 5p states in the band structure. The defect states associated with In are successfully mixed with the O 2p states without creating isolated states in the band gap. Increasing In doping level induced no substantial change in the absorption spectra. The creation of oxygen vacancy in the Indium doped TiO_2_ blue shifted the absorption edge and declined the UV light absorption. The experimentally observed improved photoactivity of In-TiO_2_ could be reasonably explained by the 2.08% In doped TiO_2_ model, which exhibits stable configuration, and reduced band gap. The proper oxidation state of In dopant stabilized the TiO_2_ system while inducing visible light absorption spectrum. Stabilizing the visible light active In doped TiO_2_ would improve its efficiency in photoelectrochemical applications.

## Figures and Tables

**Table 1:** Bond lengths (averaged) of the simulated models.

Bond Length	TiO_2_	Ti_16-1_In_1_O_32_	Ti_16-2_In_2_O_32_	Ti_16-2_In_2_O_31_
D_O-Ti_ (Å)	1.9651	1.9721	1.9846	1.9755
D_O-O_ (Å)	2.7034	2.7152	2.6886	2.7547
D_O-In_ (Å)	---	2.1294	2.1707	2.1723

**Table 2:** Band edge and Fermi level positions in different systems. The valence band maximum and conduction band minimum are represented by VBM and CBM, respectively.

	TiO_2_	Ti_16-1_In_1_O_32_	Ti_16-2_In_2_O_32_	Ti_16-2_In_2_O_31_
Fermi level (eV)	Above VBM	0.25 eV above VBM	0.1 eV below VBM	0.1 eV above VBM
VBM (eV)	0	−0.75	0.1	−0.92
CBM (eV)	2.13	1.85	2.75	2.40
